# Induction of cell proliferation and survival genes by estradiol-repressed microRNAs in breast cancer cells

**DOI:** 10.1186/1471-2407-12-29

**Published:** 2012-01-20

**Authors:** Xinfeng Yu, Xuemei Zhang, Ishwori B Dhakal, Marjorie Beggs, Susan Kadlubar, Dali Luo

**Affiliations:** 1 Department of Pharmacology, School of Chemical Biology & Pharmaceutical Sciences, Capital Medical University, 100069, Beijing, China; 2 Institute of Molecular Genetics, College of life science, Hebei United University, TangShan, 063000, China; 3 Department of Medical Genetics, College of Medicine, University of Arkansas for Medical Sciences, Little Rock, 72205 USA

## Abstract

**Background:**

In estrogen responsive MCF-7 cells, estradiol (E_2_) binding to ERα leads to transcriptional regulation of genes involved in the control of cell proliferation and survival. MicroRNAs (miRNAs) have emerged as key post-transcriptional regulators of gene expression. The aim of this study was to explore whether miRNAs were involved in hormonally regulated expression of estrogen responsive genes.

**Methods:**

Western blot and QPCR were used to determine the expression of estrogen responsive genes and miRNAs respectively. Target gene expression regulated by miRNAs was validated by luciferase reporter assays and transfection of miRNA mimics or inhibitors. Cell proliferation was evaluated by MTS assay.

**Results:**

E_2 _significantly induced bcl-2, cyclin D1 and survivin expression by suppressing the levels of a panel of miRNAs (miR-16, miR-143, miR-203) in MCF-7 cells. MiRNA transfection and luciferase assay confirmed that bcl-2 was regulated by miR-16 and miR-143, cyclinD1 was modulated by miR-16. Importantly, survivin was found to be targeted by miR-16, miR-143, miR-203. The regulatory effect of E_2 _can be either abrogated by anti-estrogen ICI 182, 780 and raloxifene pretreatment, or impaired by ERα siRNA, indicating the regulation is dependent on ERα. In order to investigate the functional significance of these miRNAs in estrogen responsive cells, miRNAs mimics were transfected into MCF-7 cells. It revealed that overexpression of these miRNAs significantly inhibited E_2_-induced cell proliferation. Further study of the expression of the miRNAs indicated that miR-16, miR-143 and miR-203 were highly expressed in triple positive breast cancer tissues, suggesting a potential tumor suppressing effect of these miRNAs in ER positive breast cancer.

**Conclusions:**

These results demonstrate that E_2 _induces bcl-2, cyclin D1 and survivin by orchestrating the coordinate downregulation of a panel of miRNAs. In turn, the miRNAs manifest growth suppressive effects and control cell proliferation in response to E_2_. This sheds a new insight into the integral post-transcriptional regulation of cell proliferation and survival genes by miRNAs, a potential therapeutic option for breast cancer.

## Background

17-β-estradiol (E_2_) regulates genes directly by binding to estrogen receptors (ERs) that are ligand-activated transcription factors and indirectly by activating plasma membrane-associated ERs which, in turn, activates intracellular signaling cascades leading to altered gene expression [1]. Therefore, ERs may participate in both the genomic (transcriptional) and non-genomic actions of E_2 _[2]. E_2_-liganded ERs interacts directly with a specific DNA sequence called the estrogen response element (ERE = 5'-AGGTCAnnnTGACCT-3') located in the promoter region of target genes [3]. DNA bound ERs then recruits transcriptional coregulators or interacts with other transcription factors, such as AP-1[4] and Sp-1 [5] to indirectly modulate target gene transcription.

To date, two isoforms of the ERs (α and β) have been identified which are able to bind to DNA as homo- or heterodimers. However, it has been shown that, in MCF-7 cells, ERα represents the predominant form, while ERβ is barely detectable [6]. Most studies so far have focused on E_2_-ERα mediated transcriptional regulation of genes involved in the control of cell proliferation and survival. It has been reported that E_2 _up-regulates the bcl-2 mRNA level in MCF-7 cells via two EREs located within the coding region [7]. The expression of cyclin D1, a gene involved in G1 phase cell cycle progression, is induced by E_2 _in human breast cancer cells. Further studies have identified multiple enhancer elements involved in this regulation [8-11]. E_2 _also induces survivin upregulation as shown by a gene expression profiling analysis [12]. In hormone-responsive human breast cancer cells, ligand-activated ERα regulates target gene transcription by binding to their DNA response elements (EREs) or by tethering to other trans-acting factors [13,14]. However, the effect of E_2 _on gene expression at the post-transcriptional level still needs further investigation.

MicroRNAs (miRNAs) are a class of evolutionarily conserved small, non-coding RNAs that control gene expression at the post-transcriptional level [15]. They regulate gene expression by base pairing to the 3'UTR of target mRNA, resulting in direct cleavage and/or translation inhibition of the target mRNA [16,17]. Several studies on miRNA array analysis in MCF-7 cells have demonstrated that E_2 _regulates a variety of miRNAs. E_2 _upregulates 21 miRNAs and downregulated 7 miRNAs in MCF-7 vector control stable cells treated with E_2 _for 4 h [18]. E_2 _downregulates the expression of mature miRNAs and pre-miRNAs (miR-195, miR-125a, miR-143, miR-145, miR-16, miR-190), but not pri-miRNAs in both mice and cells [19]. Maillot et al. [20] have shown the expression of a broad set of miRNAs (miR-181a, miR-21, miR-26a, miR-200c, miR-27b, miR-23b) decreases following E_2 _treatment in an ER-dependent manner. Based on previous microRNA expression profilings, we demonstrated that miR-16, miR-143 and miR-203 were potentially suppressed in response to E_2 _treatment in MCF-7 cells by QPCR quantification. Recently, estradiol-regulated miRNAs have been reported to control estrogen response and cell growth in breast cancer cells [18,20]. However, whether these estradiol-repressible miRNAs coordinately control cell proliferation and survival by targeting bcl-2, cyclin D1 and survivin at the post-transcriptional level in breast cancer cells is not fully investigated.

In the present study, we demonstrated that E_2 _significantly induced bcl-2, cyclin D1 and survivin expression by suppressing the expression of a set of miRNAs in ERα dependent manner in MCF-7 cells. The downregulated miRNAs exhibited the growth suppressive effect in response to E_2 _and were highly expressed in triple positive breast cancer tissues. The study revealed the post-transcriptional regulation of estradiol-induced cell proliferation and survival genes by coordinately suppressing a panel of miRNAs, which may serve as therapeutic options in breast cancer treatment.

## Methods

### Cells and treatment

17β-estradiol, raloxifene, fulvestrant (ICI 182, 780) were purchased from Sigma (Sigma, St. Louis, MO). The breast cancer cells MCF-7, MDA-MB-231 were obtained from the American Type Culture Collection (ATCC, Rockville, MD) and were maintained at 37°C under 5% CO_2_. MCF-7 cells were maintained in IMEM medium supplemented with 10% fetal bovine serum (FBS) and 0.01 mg/ml bovine insulin (Invitrogen, Carlsbad, CA). MDA-MB-231 were maintained in DMEM medium with 10% FBS. Prior to ligand treatment, cells were incubated with phenol red-free IMEM supplemented with 5% charcoal stripped FBS for 48 h (serum-starved). Then cells were treated with ethanol (vehicle control, 0.01% final volume), 10 nM E_2 _for each time course or pretreated with 1 μM ICI 182, 780 or raloxifene respectively for 6 h, then treated in combination with 10 nM E_2 _for 48 h.

### Western blot

Cells were harvested and whole cell extracts were prepared in modified RIPA buffer and separated by 4-12% NuPAGE Bis-Tris gel electrophoresis (Invitrogen, Carlsbad, CA). Proteins were transferred onto PVDF membrane and probed with anti-cyclin D1, anti-bcl-2 (Santa Cruz Biotechnology, Santa Cruz, CA), anti-survivin (R&D systems, Minneapolis, MN) antibodies at 4°C overnight, then the membrane was incubated with secondary antibody for 1 h before chemiluminescence detection using Pierce ECL Western Blotting Substrate (Pierce, Rockford, IL). β-actin was also detected as a loading control using mouse monoclonal antibody (Sigma, St. Louis, MO).

### Transfection of siRNA and miRNA

MiR-16, miR-143, miR-203 mimics and inhibitors were purchased from Ambion and transfected into cells with lipofectamine 2000 (Invitrogen, Carlsbad, CA). The final concentration of miR-16, miR-143, miR-203 mimics and inhibitors was 40 nM. After 48 h, cells were harvested and bcl-2, cyclin D1 and survivin expression were measured. A nonspecific miRNA mimic or inhibitor was used as negative control.

ERα siRNA was purchased from Dharmacon SMARTpool siRNA, MCF-7 cells were transfected with 100 nM ERα siRNA for 24 h using lipofectamine 2000, then stimulated with 10 nM E_2 _for 24 h and 48 h.

### Real-time quantitative PCR (QPCR)

For detection of miRNA expression, miScript Reverse Transcription Kit (Qiagen, Valencia, CA) was used for cDNA synthesis. miScript SYBR Green PCR Kit (Qiagen, Valencia, CA), in combination with a pair of miRNA specific primers were used for mature miRNA detection. RNU6B was used as an internal control. Taqman probes and gene specific primers for FAM-labeled ERα and VIC-labeled β-actin were obtained from Applied Biosystem (Foster City, CA) and PCR condition for determination of ERα mRNA level was described previously [21]. Relative gene expression was determined using a previously described method [22].

### Plasmid construction and luciferase assay

To make luciferase constructs containing 3' UTR of *survivin*, 3'UTR was amplified using a pair of primers whose sequences were 5'-gcTCTAGActgcctggtcccagagtg-3'and 5'-gcTCTAGAtaaaaccacatgagactttattgg-3'. PCR was performed with genomic DNA and digested using *Xba *I and ligated into pGL3-control vector (Promega Corporation, Madison, WI). The constructs were sequenced to ascertain the right orientation and authenticity in the vector. The 3' UTR of *bcl-2 *and *cyclin D1 *cloned into *Xba *I site of pGL-3 promoter were kindly provided by Dr. Ruggero De Maria (Mediterranean Institute of Oncology, Catania, Italy).

Cells were plated in a 96-well plate and grown to 80-90% confluence. The firefly luciferase constructs (100 ng) were cotransfected with 40 nM miRNA mimics into MCF-7 cells using lipofectamine 2000 reagent. To monitor transfection efficiency, cells were cotransfected with 10 ng of the pRL-SV40 plasmid which encodes Renilla luciferase. Luminescence was measured 24 h after transfection using a dual-luciferase reporter assay system (Promega Corporation, Madison, WI). All transfections were performed in triplicate, and data were analyzed by normalizing firefly luciferase activity to Renilla luciferase activity for each sample. Each construct was tested in three independent transfections.

### MTS Cell proliferation assay

MCF-7 cells were incubated in 5% charcoal stripped FBS for 24 h, then transfected with 40 nM negative control or miR-143, miR-16 and miR-203 mimics. After 24 h, cells were trypsinized into 96-well plates. Cells were treated with 10 nM E_2 _or ethanol (vehicle control) for 5 days, CellTiter 96 AQueous One Solution Cell Proliferation Assay (MTS) (Promega Corporation, Madison, WI) was performed to detect cell proliferation.

### Triple positive and negative breast cancer tissues

Fourteen triple positive and 36 triple negative formalin-fixed paraffin-embedded (FFPE) breast cancer tissue specimens were obtained from Bioserve Global Biorepository (Beltsville, MD). RNA was isolated from these FFPE tissue specimens using miRNeasy FFPE kit (Qiagen, Valencia, CA) for detection of miR-16, miR-143 and miR-203 expression in triple positive and triple negative breast samples. MiR-631, a non-estradiol inducible miRNA was used as control. These studies were approved by the Institutional Review Board at University of Arkansas for Medical Sciences.

### Statistical analysis

The differential analysis per miRNA expression was performed using a 2-sample (treated vs. control and triple positive vs. negative samples) Student's *t*-test. MicroRNAs expression was normalized with respect to RNU6B as an internal control. All statistical analyses were performed using the SAS software (version 9.1; SAS Institute, Inc., Cary, NC). A *P *value of less than 0.05 (2-sided) was considered to be statistically significant.

## Results

### Identification of estradiol induced cell proliferation and survival genes in MCF-7 cells

**Figure 1:**
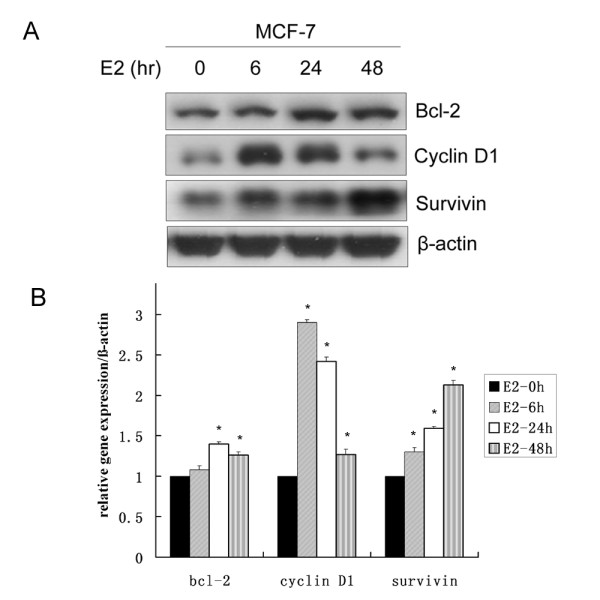
**E_2 _induced bcl-2, cyclin D1 and survivin expression in MCF-7 cells**. (**A**) MCF-7 cells were incubated with phenol red-free IMEM supplemented with 5% charcoal stripped FBS for 48 h (serum-starved). Then cells were treated with vehicle control or 10 nM E_2 _for 6, 24 and 48 h, total protein was extracted to detect the expression of bcl-2, cyclin D1 and survivin by Western blot. (**B**) The densitometry of each gene vs. β-actin was indicated and the statistical analysis was shown. * denotes *P *< 0.05 compared with vehicle control.

ER-positive MCF-7 cells have been extensively used as a model of hormone-dependent breast cancer. In response to E_2 _stimulation, MCF-7 cells exhibit cell proliferation and growth response in an E_2 _dependent manner. Bcl-2, cyclin D1 and survivin have been shown to be implicated in the process of cell proliferation and survival. In previous studies, bcl-2 [7,23,24] and cyclin D1[9,10,25] mRNA levels have been found to be upregulated by E_2_. Yet very few studies on the regulation of survivin by E_2 _[12]. Consistently, we found that E_2 _significantly induced cyclin D1 expression at 6 h and 24 h, it also dramatically enhanced bcl-2 and survivin expression at 24 h and 48 h (Figure 1). The upregulation of bcl-2, cyclin D1 and survivin induced by E_2 _was in a time-dependent manner.

### Downregulation of a panel of miRNAs induced by E_2_

**Figure 2:**
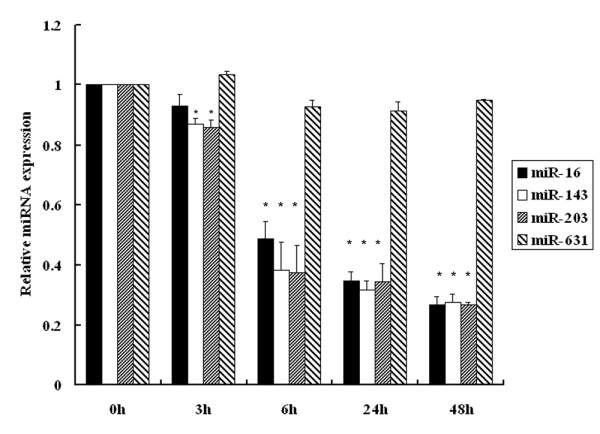
**E**_2 _**reduced miR-16, miR-143 and miR-203 expression in MCF-7 cells**. MCF-7 cells were treated as described in Figure 1 and stimulated by E_2 _for 3, 6, 24 and 48 h. Total RNA was extracted and miRNA expression levels were examined by QPCR. MiR-631 is a non-E_2 _responsive miRNA serving as negative control. * denotes *P *< 0.05 compared with vehicle control.

ERα-mediated transcriptional regulation is one of the mechanisms of gene upregulation induced by E_2_. MiRNAs have recently been shown to be another important post-transcriptional regulation of genes involved in cell growth and estradiol response in MCF-7 cells [18,26,27]. In order to explore whether miRNAs participate in the upregulation of bcl-2, cyclin D1 and survivin induced by E_2_, we focused on the downregulated miRNAs that could play a role in the modulation of cell proliferation and survival genes at the post-transcriptional level. Based on previous miRNA array profilings [18,20], QPCR was performed to examine the expression of miRNAs and it showed that miR-143, miR-16 and miR-203 were robustly repressed at 6, 24 and 48 h upon E_2 _treatment (Figure 2) miR-631, a non-estradiol inducible miRNA, was used as a control in response to E_2 _treatment.

T47D is another estrogen responsive ERα positive breast cancer cell line which is used to examine the expression of bcl-2, cyclinD1 and survivin in response to E_2 _treatment. Consistently, E_2 _enhanced the expression of these genes but moderately reduced the expression of miR-16, miR-143 and miR-203 as shown in Additional file 1: Figure S1. Therefore, T47D cells displayed a similar effect in response to E_2 _stimulation, although the effect was weaker than that of MCF-7 cells.

### MiRNAs regulate bcl-2, cyclin D1 and survivin at the post-transcriptional level

**Figure 3:**
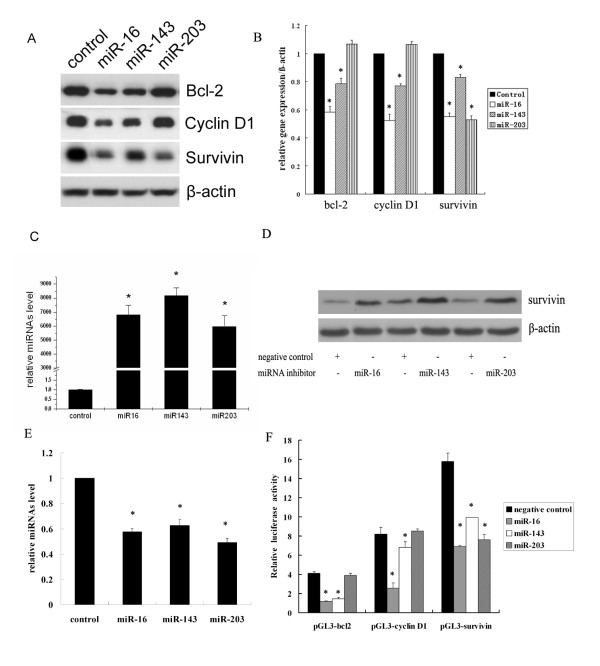
**E_2_-responsive miRNAs downregulated endogenous bcl-2, cyclin D1 and survivin expression**. **(A) **MCF-7 cells were transfected with 40 nM miR-16, miR-143 and miR-203 mimics for 48 h, Western blot was performed to detect endogenous bcl-2, cyclin D1 and survivin expression. (**B**) The densitometry of each gene vs. β-actin was indicated and statistically analyzed. * denotes *P *< 0.05 compared with negative control miRNA. (**C**) RNA was extracted from MCF-7 transfected with miRNA mimics and RT-QPCR was performed to confirm the overexpression of these miRNAs. (**D, E**) MCF-7 cells were transfected with 40 nM miR-16, miR-143 and miR-203 inhibitors. After 48 h, Western blot was performed to examine endogenous survivin expression. Meanwhile, RT-QPCR was performed to examine the miRNAs expression. (**F**) MCF-7 cells were co-transfected with 40 nM miR-16, miR-143 and miR-203 mimics together with 100 ng bcl-2, cyclin D1 and survivin 3'UTR luciferase constructs and 10 ng pRL-SV40. After 24 h, luciferase activity assay was determined. * denotes *P *< 0.05 compared with negative control.

E_2 _treatment leads to an increase in cell proliferation and survival genes bcl-2, cyclin D1 and survivin, it can also reduce the expression of several miRNAs. So we hypothesize that E_2 _upregulates target genes associated with cell proliferation and survival by coordinately suppressing the expression of miR-143, miR-16 and miR-203. It has been clearly demonstrated that miR-15 and miR-16 cluster targets bcl-2 and cyclin D1 [28,29] and miR-143 targets bcl-2 [30]. Our results were in accordance with previous studies as shown in Figure 3A and 3B. These miRNAs were able to significantly downregulate endogenous target genes bcl-2 and cyclin D1 expression at the protein level. QPCR was used to examine the level of the miRNAs and it indicated that transfection of miRNA mimics remarkably increased the level of miRNAs (Figure 3C). Furthermore, TargetScan and miRbase programs predict conserved binding sites of miR-16, miR-143 and miR-203 in the 3'-UTR of survivin. We further confirmed that these miRNAs target survivin at the post-transcriptional level by transfection of miRNA inhibitors and subsequent luciferase assay. It has been shown that transfection of miRNA inhibitors can potentially interfere with the expression of endogenous miRNAs and therefore enhance the expression of target gene survivin (Figure 3D and 3E).

The biological activity of miRNAs is primarily mediated by interaction with binding sites in the 3'-UTR of target genes and translational inhibition. Finally, luciferase assay was performed to confirm the binding of the miRNAs to the 3'UTR of target genes. MCF-7 cells were co-transfected with miR-16, miR-143 or miR-203 mimics with bcl-2, cyclin D1, survivin 3'-UTR luciferase constructs containing the miRNAs binding sites respectively. As expected, miR-16 significantly suppressed the luciferase activity of bcl-2, cyclin D1, and survivin whereas miR-143 moderately inhibited luciferase activity of bcl-2 and survivin. Noticeably, miR-203 impaired luciferase activity of survivin. Therefore, the luciferase assay revealed that these miRNAs directly regulate the expression of the target genes bcl-2, cyclin D1, and survivin by binding to the 3'UTR of these genes (Figure 3F). Based on our previous results, it is likely that E_2_-mediated reduction in miRNAs expression resulted in lower amounts of miRNAs available to bind to their recognition sequences of bcl-2, cyclin D1 and survivin and thus increasing the luciferase activity of the reporter transcripts. This is possibly one mechanism of E_2 _induced upregulation of genes involved in cell proliferation.

### Regulation of miRNAs and the expression of target genes are ERα-dependent

**Figure 4:**
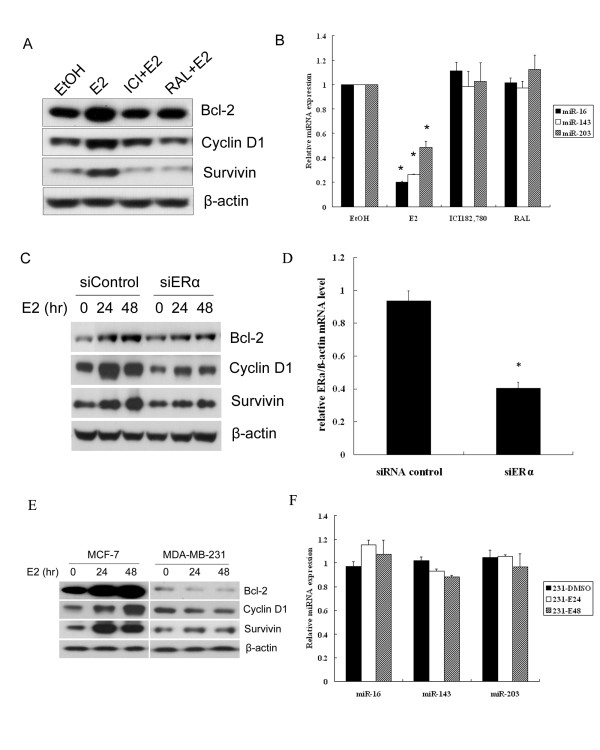
**Regulation of miRNAs and the expression of target genes are ERα-dependent**. **(A, B) **MCF-7 cells were pretreated with 1 μM ICI 182, 780 or Raloxifene for 6 h, then treated with 10 nM E_2 _for 48 h, protein was used for detection of bcl-2, cyclinD1 and survivin expression by Western blot. RNA was used to detect the expression of miRNAs by QPCR. * denotes *P *< 0.05 compared with vehicle control (ethanol). **(C, D) **MCF-7 cells were transfected with 100 nM ERα siRNA for 24 h, then cells were treated with 10 nM E_2 _for 24 h and 48 h. Western blot was used to detect the expression of bcl-2, cyclin D1 and survivin. QPCR was used to examine the level of ERα mRNA. **(E, F) **MDA-MB-231 cells were treated with 10 nM E_2 _for 24 and 48 h, miRNAs and target genes were examined. MCF-7 cells were used as ER-positive control.

To investigate whether E_2_-mediated miRNA reduction and upregulation of target genes can be abrogated by anti-estrogens, we used ICI 182, 780 and raloxifene as antagonists of E_2_. ICI 182, 780, a pure antiestrogen, is a competitive antagonist of E_2 _and blocks the transcriptional activation properties of ERs [31,32]. Raloxifene belongs to the family of selective estrogen receptor modulators (SERMs) that display agonistic or antagonistic activity in a tissue-dependent manner [33]. As we have shown, both ICI 182, 780 and raloxifene can attenuate the reduction of the miRNAs and consequently the induction of bcl-2, cyclin D1 and survivin expression by E_2 _(Figure 4A, B).

E_2 _binds predominantly to ERα which leads to transcriptional regulation of genes involved in the control of cell growth and survival. To explore whether regulation of miRNA and the target genes is ERα-dependent, siRNA was used to interfere with the expression of ERα. It was found that knockdown of ERα remarkably impaired the induction of bcl-2, cyclin D1 and survivin by E_2 _(Figure 4C, D). In addition, E_2_-regulated cell proliferation and survival genes were examined in ER-negative MDA-MB-231 cells compared with those of MCF-7 cells. In contrast, E_2 _could not enhance bcl-2, cyclinD1 and survivin expression in MDA-MB-231 cells (Figure 4E). Further studies on miRNA expression indicated that miR-16, miR-143 and miR-203 were not significantly affected by E_2 _in MDA-MB-231 cells (Figure 4F), indicating the potential modulation of cell growth and survival genes by miRNAs in reponse to E_2 _was mediated by ERα.

### MiR-16, miR-143 and miR-203 suppress E_2_-dependent cell proliferation

**Figure 5:**
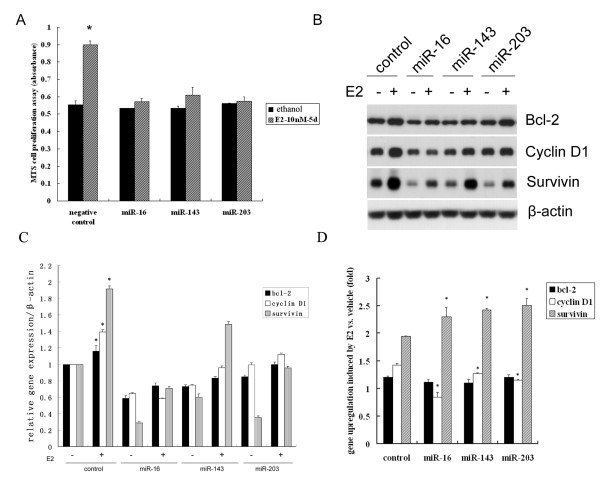
**MiRNAs inhibit E**_2_**-induced cell proliferation**. MCF-7 cells were transfected with 40 nM miR-16, miR-143 and miR-203 and treated with 10 nM E_2 _for 5 days. **(A) **MTS assay was used to detect cell proliferation. * denotes *P *< 0.05 compared with vehicle control. **(B) **MCF-7 cells were transfected with 40 nM miR-16, miR-143 and miR-203 and treated with 10 nM E_2 _for 48 h. Western blot was used to detect bcl-2, cyclin D1 and survivin expression. (**C**) The densitometry of each gene vs. β-actin was indicated. In negative control miRNA group, E_2 _significantly induced upregulation of bcl-2, cyclin D1 and survivin compared with vehicle control (* denotes *P *< 0.05). (**D**) In each miRNA-transfected group, the expression of bcl-2, cyclin D1 and survivin induced by E_2 _was normalized by that of vehicle control (ethanol). The fold change was statistically analyzed compared with negative control miRNA group. * denotes *P *< 0.05 compared with control.

E_2 _functions as a mitogen to stimulate cell proliferation and cell cycle transition. In response to E_2 _treatment, miR-143, miR-16 and miR-203 were repressed and their target genes bcl-2, cyclinD1 and survivin were upregulated. We therefore investigated whether overexpression of miR-143, miR-16 and miR-203 interfered with E_2_-induced cell proliferation in MCF-7 cells. The MTS assay showed that transfection of miR-143, miR-16 and miR-203 significantly inhibited E_2_-induced cell proliferation (Figure 5A). Since miRNAs regulated bcl-2, cyclin D1 and survivin at the endogenous level as shown in Figure 3A, we further explored whether transfection of these miRNAs impaired E_2_-induced upregulation of bcl-2, cyclin D1 and survivin that control cell proliferation and cell cycle transition. As shown in Figure 5B and 5C, Western blot and densitometry were performed to determine the protein levels of these genes. In negative control miRNA group, E_2 _significantly induced upregulation of bcl-2, cyclin D1 and survivin compared with vehicle control. Figure 5D depicted the statistical analysis of the gene expression based on the results from Figure 5C. The miRNAs significantly inhibited E_2_-induced cyclin D1 upregulation, in particular miR-16 inhibited E_2 _induced upregulation of cyclin D1 by 41% compared with negative control miRNA group. But the miRNAs have little inhibitory effects on E_2_-induced bcl-2 and no inhibitory effects on E_2_-induced survivin at all when compared with negative control miRNA group (Figure 5D). This probably was due to the robust interference of endogenous genes by the miRNAs in each group, which was in agreement with the result of Figure 3A. More importantly, these miRNAs impaired E_2_-induced gene upregulation when compared with E_2_-induced negative control miRNA group. The data indicated that miR-143, miR-16 and miR-203 not only inhibited endogenous expression of bcl-2, cyclinD1 and survivin, but also disturbed E_2_-induced upregulation of cyclin D1, an important cell cycle regulator, thereby, accounting for the impairment of cell proliferation.,

### MiR-16, miR-143 and miR-203 are highly expressed in ER positive breast tumors

**Figure 6:**
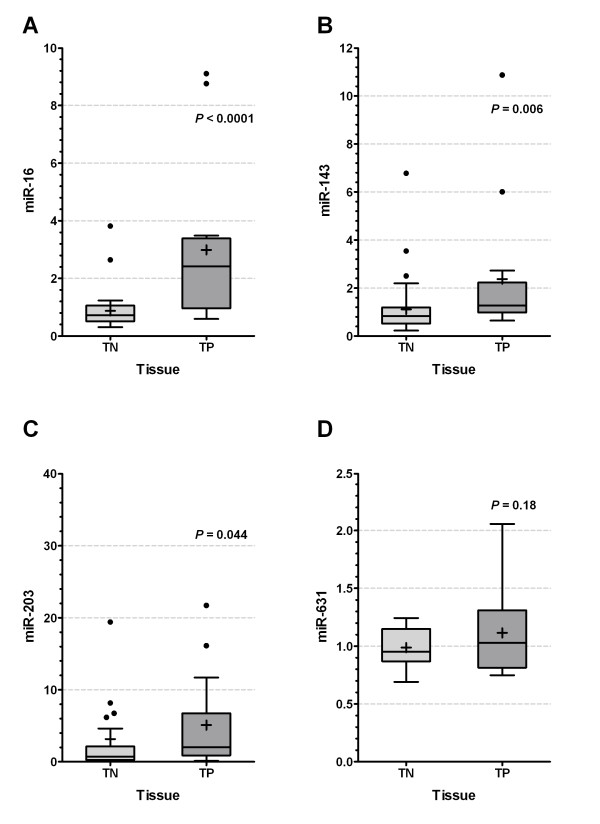
**miRNAs were highly expressed in triple positive breast cancer tissues**. **(A-D) **Total RNA was extracted from 14 triple negative and 36 triple positive breast cancer tissues to detect miRNAs expression by QPCR. MicroRNAs expression was normalized with respect to RNU6B as an internal control. *T*-test was used to indicate the difference. *P *values are shown in the figures. TP denotes triple positive breast samples, TN means triple negative breast samples.

Triple-negative and triple positive breast cancers are defined by the status of estrogen receptor (ER), progesterone receptor (PR) and HER-2 expression. Because the triple-negative phenotype is more aggressive and not amenable to any form of endocrine therapy and has a high incidence of metastasis, the patients have a worse prognosis than patients with the triple positive phenotype [8]. Since miR-16, miR-143 and miR-203 target genes control cell proliferation and the miRNAs exhibit a growth suppressive effect in response to E_2_, we speculated that these miRNAs may be differentially expressed in ER positive and negative breast tumors, acting as a potential causal link with tumor suppressive effects in breast cancer progression. Notably, as shown in Figure 6, miR-16, miR-143 and miR-203 were highly expressed in ER positive breast tumor, in contrast, miR-631 was not differentially expressed between ER positive and negative breast cancer, suggesting these anti-onco miRNAs may play an important role in breast cancer progression and response to chemotherapy.

## Discussion

The discovery of miRNAs as a novel class of gene expression regulators has provided new strategies for disease diagnostics and therapeutics. Cell cycle, cell proliferation, cell survival and tumorigenesis are all regulated by miRNAs. Altered abundance of cell survival and cell cycle regulation proteins and aberrant expression of miRNAs frequently coexist in human breast cancers [34]. miRNAs are aberrantly expressed or mutated in cancer, acting as a novel class of oncogenes or tumor suppressor genes [35]. In this study, we demonstrated that a set of E_2_-repressible microRNAs in breast cancer cell lines was associated with altered cell cycle progression and cell proliferation, which could play a causal role for miRNAs in controlling breast tumor growth.

In breast cancer, abnormalities of the cell cycle are frequently observed in response to E_2 _treatment. Cyclin D1 encodes a key regulator of the cell cycle transition from G1 to S phase and is overexpressed in more than 50% of breast cancers, functioning as a rate-limiting factor for human breast cancer cell proliferation *in vivo *and *in vitro *[36,37]. The bcl-2 protein is associated with the inner mitochondrial membrane and functions to inhibit apoptosis and promote survival [38,39]. Survivin, a member of the inhibitor of apoptosis (IAP) family of antiapoptotic proteins, regulates the G1 checkpoint and G2/M phase of the cell cycle by associating with mitotic spindle microtubules. Survivin directly inhibits caspase-3 and caspase-7 activity, is inversely correlated with apoptosis and is positively correlated with cell proliferation [40,41]. We demonstrated that E_2 _induced upregulation of cyclin D1, bcl-2 and survivin in MCF-7 cells, which played an important role in E_2 _stimulated cell proliferation and cell cycle transition. This result was consistent with previous studies [7,9,10,23-25]. Similar results were also observed in another estrogen-responsive breast cancer cells T47D, E_2 _dramatically induced the expression of cyclin D1, bcl-2 and survivin as shown in Additional file 1: Figure S1.

MiRNAs have emerged as a novel regulator of gene expression at the post-transcriptional level by base-pairing interactions between miRNAs and the 3'-UTR of their target mRNAs [16]. In order to explore whether miRNAs participate in the upregulation of bcl-2, cyclin D1 and survivin by E_2_, we focused on the E_2_-repressible miRNAs that may play a role in the modulation of cell proliferation and survival genes. Several studies have demonstrated that E_2 _upregulates or downregulates a variety of miRNAs by miRNA expression profilings in MCF-7 cells [18-20]. The difference of the results may be due to the cell status, treatment and stimulation time. Based on previous studies, we demonstrated miR-16, miR-143 and miR-203 were coordinately suppressed in response to E_2 _treatment using QPCR quantification. Therefore, we proposed that these miRNAs might be involved in the regulation of cell proliferation and survival by targeting bcl-2, cyclin D1 and survivin at the post-transcriptional level.

Some publications have provided support for our hypothesis, Cimmino et al. [28] have demonstrated that *miR-15a *and *miR-16 *expression is inversely correlated with bcl-2 expression in Chronic lymphocytic leukemia and that both microRNAs negatively regulate bcl-2 at a post-transcriptional level. Bonci et al. [29] have demonstrated that the miR-15a-miR-16 cluster targets CCND1 (encoding cyclin D1), acting as tumor suppressor genes in prostate cancer by the control of cell survival, proliferation and invasion. miR-143 has been validated to target the oncogene KRAS [42] and may also modulate extracellular-regulated protein kinase 5 (ERK5) [43,44] and bcl-2 [30]. In this study, we confirmed the previous studies that miR-16 targets bcl-2 and cyclin D1 and miR-143 targets bcl-2. However, we can also see the impairment of endogenous cyclin D1 by miR-143 as shown in Figure 3A, which may result from the interference of ERK5 expression by miR-143 [30], since ERK5 has been shown to regulate cyclin D1[45,46]. TargetScan and miRbase programs predict conserved binding sites of miR-16, miR-143 and miR-203 in 3'-UTR of survivin. We further elucidated that miR-16, miR-143 and miR-203 target survivin at the post-transcriptional level by transfection of miRNA mimics and inhibitors. Luciferase activity assay indicated these miRNA directly regulated the expression of survivin by binding to the 3'-UTR of survivin.

A miRNA regulates a variety of target genes, and a gene is modulated by many miRNAs. Therefore, in response to E_2 _stimulation, several miRNAs are coordinately suppressed to upregulate the target genes which are involved in cell proliferation and survival. It provides a novel mechanism for regulation of genes containing ERE in the promoters. Previous studies have shown the ERα-mediated transcriptional regulation of bcl-2, cyclin D1 mRNAs by binding to the ERE of target genes. We cannot conclude that the increase of bcl-2, cyclin D1 and survivin is due solely to E_2_-mediated reduction of miR-16, miR-143 and miR-203, but miRNAs do play a crucial role in the post-transcriptional regulation because when actinomycin D was used to inhibit *de novo *RNA synthesis, E_2 _can still enhance the expression of bcl-2, cyclinD1 and survivin, though at a low level (Additional file 2: Figure S2). Further studies will be needed to dissect the relative contributions of ERα-mediated multiple pathways controlling bcl-2, cyclin D1 and survivin expression.

ERα is essential for E_2_-dependent growth, and its level of expression is a crucial determinant of response to endocrine therapy and prognosis in ERα-positive breast cancer. Our data indicated that ICI 182, 780 and Raloxifene can abrogate E_2 _repressed miRNA levels and therefore attenuate the expression of target genes. It is reported that ICI 182, 780 and Raloxifene can locally alter the ERα ligand binding structure via specific hydrophobic residues and decrease its transcriptional activity [47]. In addition, knockdown of the ERα protein also impaired E_2 _induced upregulation of bcl-2, cyclin D1 and survivin. In ERα negative MDA-MB-231 cells, E_2 _has no effect on the regulation of miRNAs and target genes as shown in Figure 4E and 4F. In ERα negative non-cancer cells MCF-10A, we also observed similar effect (data not shown). Thus, the regulation is mainly dependent on ERα protein expression and transactivation in both breast cancer cells and in a normal breast cells.

Suzuki et al. [48] have recently shown that a central tumor suppressor, p53, enhances the post-transcriptional maturation of several miRNAs with growth-suppressive function, including miR-16, miR-143, miR-145 and miR-203 in response to DNA damage. P53 interacts with the Drosha processing complex through the association with the DEAD-box RNA helicase p68 and facilitates the processing of primary miRNAs to precursor miRNAs. In our study, we used MCF-7 cells carrying wild type p53 genes as a model to investigate the E_2_-repressible miRNAs target genes involved in cell proliferation. However, in p53 mutant ERα-positive T47D cells, we observed similar regulation yet to a weaker extent. Previous studies have elucidated the interaction of ERα and p53 [49,50]. It is likely that both ERα and p53 participated in the regulation of E_2_-repressible miRNAs. Further studies are needed to elucidate whether ERα-mediated induction of target genes by E_2 _via p53-regulated miRNA maturation.

The downregulated miRNA exhibits the growth suppressive effect in response to E_2_. miR-16 and miR-15 act as tumor suppressors and control cell cycle transition by targeting cyclin D1 and cyclin E [29,51]. MiR-143 has been claimed to be a anti-oncomir in human colorectal tumors and can increase the sensitivity of chemotherapy [30,52-54]. MiR-203 has recently been identified to inhibit cell proliferation and invasion in prostate cancer, and reverse chemoresistance in p53-mutated colon cells [55-57]. In this study, overexpression of miR-16, miR-143 and miR-203 remarkably inhibited E_2 _induced cell proliferation of breast cancer cells. Further studies have shown that these miRNAs were significantly higher in triple positive than in triple negative breast tissues. This suggested a potentially tumor suppressive effect of the miRNAs on cancer progression of ER positive breast cancers, which may open new avenues for therapeutic intervention in breast cancer treatment.

## Conclusions

It is our novel discovery that estradiol suppressed a panel of miRNAs, involving in the coordinated modulation of target genes that control cell proliferation and survival in breast cancer progression. Importantly, these miRNAs manifest tumor suppressive effects in response to estradiol stimulation and could possibly be biomarkers in triple positive breast tumors. In addition they may be potential biomarkers of breast cancer subtypes. Uncovering the critical role of these miRNAs in tumor suppression will contribute to the efficacy of breast cancer therapy.

## Abbreviations

E_2_: 17β-estradiol; ER: Estrogen receptor; PR: Progesterone receptor; ERE: Estrogen response element; MiRNA: MicroRNA; QPCR: Real-time quantitative PCR; UTR: Untranslated region; DMEM: Dulbecco's modified eagle's medium; IMEM: Improved minimum essential medium; FBS: Fetal bovine serum; ECL: Enhanced chemiluminesence; FFPE: Formalin-fixed paraffin-embedded; SERM: Selective estrogen receptor modulators; MTS: 3-(4, 5-dimethylthiazol-2-yl)-5-(3-carboxymethoxyphenyl)-2-(4-sulfophenyl)-2H-(tetrazolium).

## Competing interests

The authors declare that they have no competing interests.

## Authors' contributions

XY designed and performed the experiments, XY and XZ drafted the manuscript. ID was responsible for data analyses. MB contributed to sample preparation. SK, MB and DL provided technical support and critically reviewed the manuscript. All authors read and approved the final manuscript.

## Acknowledgements

We thank Ruggero De Maria (Mediterranean Institute of Oncology, Catania, Italy) for the generous gift of pGL3-bcl-2 and pGL3-cyclinD1 plasmids. We thank Dr. Zhihua Liu (Chinese Academy of Medical Sciences) for the technical support. This work was supported by the funding of Susan G. Komen for the Cure BCTR0707584 and the National Natural Science Foundation (30973537).

## Pre-publication history

The pre-publication history for this paper can be accessed here:

http://www.biomedcentral.com/1471-2407/12/29/prepub

## Supplementary Material

Additional file 1 **Figure S1. E_2 _induced the upregulation of bcl-2, cyclinD1 and survivin and moderately suppressed the level of the miRNAs in T47D cells**. **(A) **T47D cells were incubated with phenol red-free IMEM supplemented with 5% charcoal stripped FBS for 48 h. Then cells were treated with vehicle control or 10 nM E_2 _for 24 and 48 h, total protein was extracted to detect the expression of bcl-2, cyclin D1 and survivin by Western blot. **(B) **RNA was extracted from the cells and RT-QPCR was used to examine the level of the miRNAs. Click here for file

Additional file 2 **Figure S2. E_2 _induced upregulation of bcl-2, cyclin D1 and survivin at both transcriptional and the post-transcriptional level**. **(A) **MCF-7 cells were pretreated or not with 2 μg/ml actinomycin D for 1 h and then stimulated with 10nM E_2 _for 12 h. Total protein was extracted to determine the expression of bcl-2, cyclin D1 and survivin. **(B) **The densitometry of each gene vs. β-actin was indicated and statistical analysis was shown. * denotes *P *< 0.05 compared with control (the first group). Click here for file
