# Supplementary figures and images for: Induction of cell proliferation and survival genes by estradiol-repressed microRNAs in breast cancer cells
Source: BMC Cancer. 2012 Jan 20;12:29. doi: 10.1186/1471-2407-12-29 (PMC3274428; doi:10.1186/1471-2407-12-29)

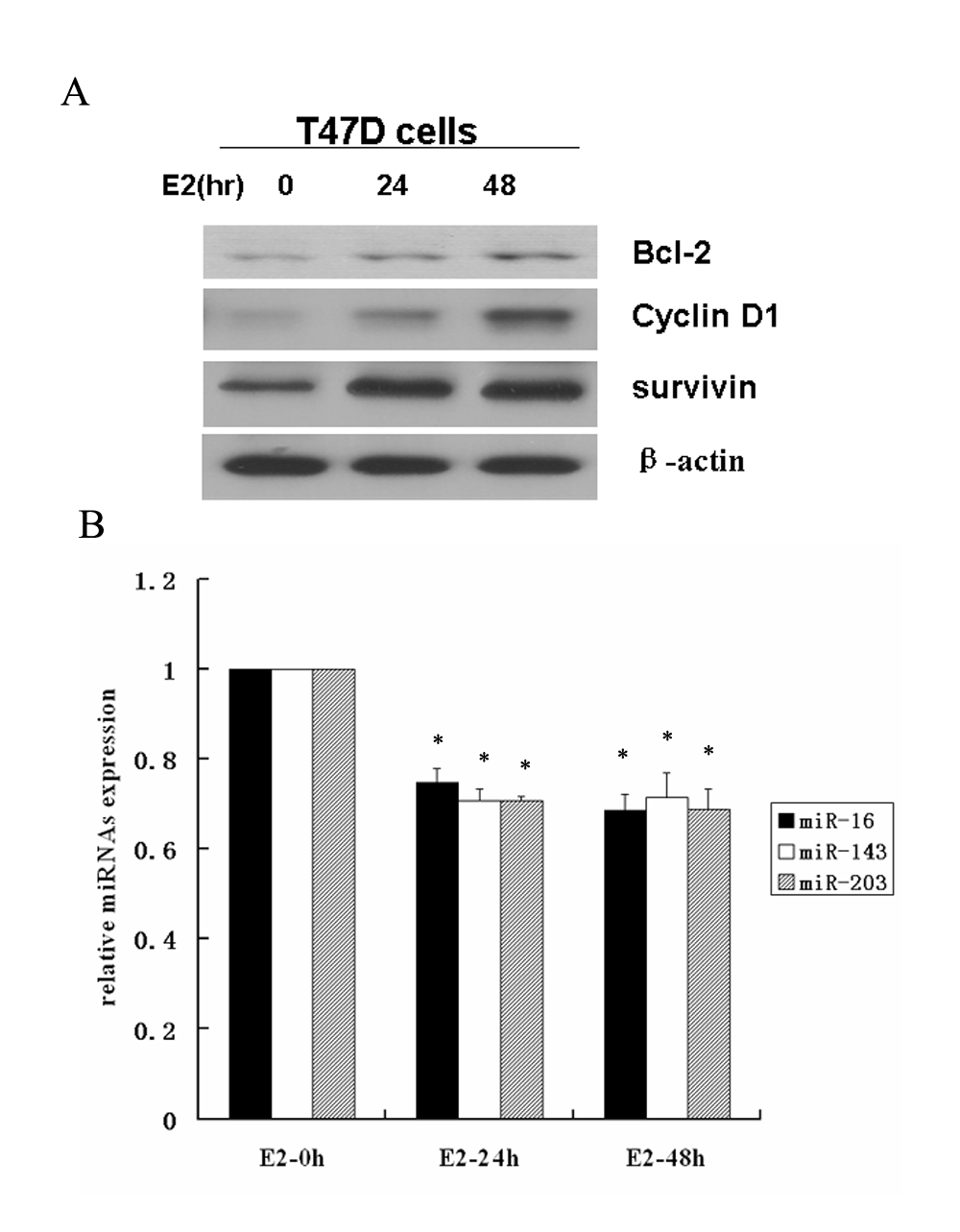

Supplement: Additional file 1 — Figure S1. E2 induced the upregulation of bcl-2, cyclinD1 and survivin and moderately suppressed the level of the miRNAs in T47D cells. (A) T47D cells were incubated with phenol red-free IMEM supplemented with 5% charcoal stripped FBS for 48 h. Then cells were treated with vehicle control or 10 nM E2 for 24 and 48 h, total protein was extracted to detect the expression of bcl-2, cyclin D1 and survivin by Western blot. (B) RNA was extracted from the cells and RT-QPCR was used to examine the level of the miRNAs. [file 1471-2407-12-29-S1.TIFF]

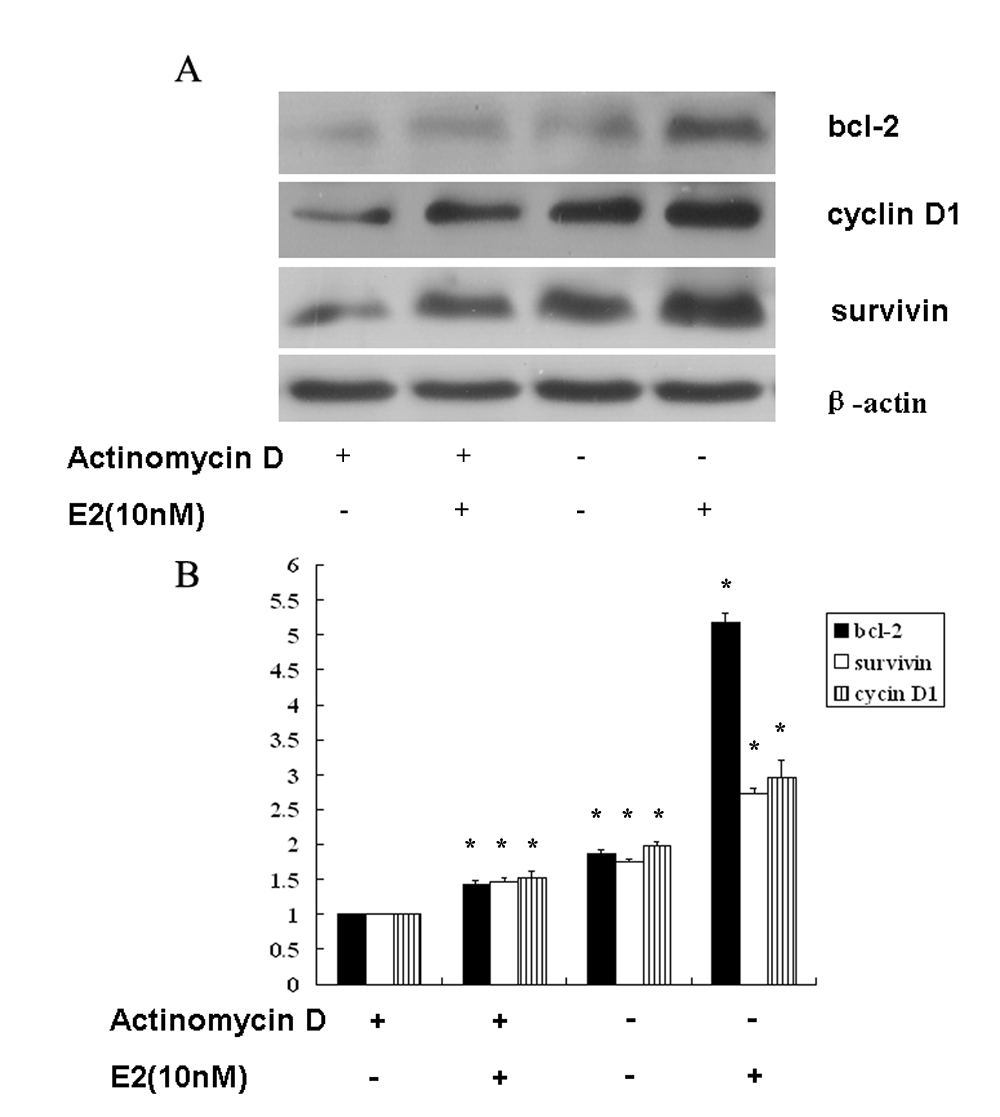

Supplement: Additional file 2 — Figure S2. E2 induced upregulation of bcl-2, cyclin D1 and survivin at both transcriptional and the post-transcriptional level. (A) MCF-7 cells were pretreated or not with 2 μg/ml actinomycin D for 1 h and then stimulated with 10nM E2 for 12 h. Total protein was extracted to determine the expression of bcl-2, cyclin D1 and survivin. (B) The densitometry of each gene vs. β-actin was indicated and statistical analysis was shown. * denotes P < 0.05 compared with control (the first group). [file 1471-2407-12-29-S2.TIFF]
